# The Burden of Respiratory Syncytial Virus Infection in Adults and Reproductive-Aged Women

**DOI:** 10.9745/GHSP-D-19-00121

**Published:** 2019-12-23

**Authors:** Bernard Gonik

**Affiliations:** a Wayne State University School of Medicine, Detroit, MI, USA.

## Abstract

Currently available data on respiratory syncytial virus (RSV) disease burden in adults and reproductive-aged women are limited. These data are critically needed to assist in the advancement of strategies related to maternal RSV vaccination for the passive protection of their newborn children.

## INTRODUCTION

In 2015, it was estimated that respiratory syncytial virus (RSV) infection resulted in 33.1 million episodes of acute lower respiratory infection worldwide, with 3.2 million hospitalizations and approximately 59,600 in-hospital related deaths.^1^ These numbers likely underestimate the burden of disease because the overwhelming majority of cases are thought to occur in developing countries where surveillance data are even more limited.^1^ Globally, RSV-associated acute lower respiratory infection is one of the leading causes of morbidity and mortality in children younger than 5 years.^1^ Recently, this pathogen has also been recognized to cause significant disease in the elderly.^2^ Despite 60 years of RSV research and vaccine exploration (Figure), there is only 1 approved intervention to prevent RSV infections. Palivizumab, a monoclonal antibody against the RSV fusion protein, is only indicated for preterm infants and children at high risk for RSV infections. No licensed vaccine currently exists. However, currently, 14 candidate vaccines are being tested in clinical trials.^4^ Active vaccination of pregnant women in the third trimester is a particularly attractive approach because the most severe disease occurs within the first 6 months of life in their progeny. With current ongoing activities, approval of the first RSV vaccine for the prevention of RSV in all infants or perhaps the elderly is likely to occur in the next 6 years.

**FIGURE.**
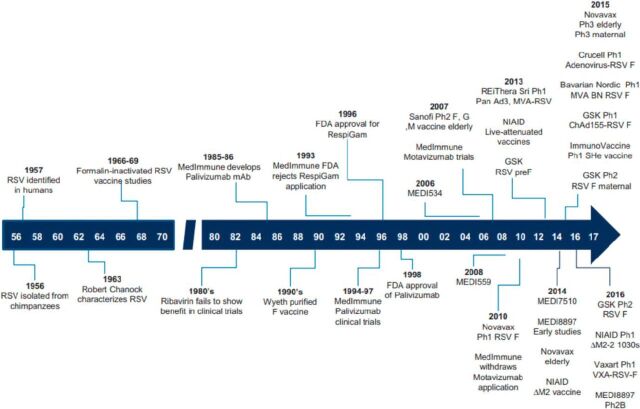
History of Respiratory Syncytial Virus Vaccine and Monoclonal Antibody Research^3^ Abbreviations: BN, Bavarian Nordic; ChAd, chimpanzee-derived adenovirus; F, respiratory syncytial virus fusion protein; FDA, United States Food and Drug Administration; GSK, Glaxo Smith Kline; mAb, monoclonal antibody; MEDI; MedImmune; MVA, modified vaccinia Ankara; NIAID, National Institute of Allergy and Infectious Diseases; PanAd, simian adenovirus; Ph, phase; RSV, respiratory syncytial virus; VXA, Vaxart.

### Why Characterize Disease Burden in a Non-Pediatric Population?

Although RSV vaccine discussion has focused on childhood infection, for several reasons, it may be important to better understand and characterize RSV disease burden in women of childbearing age. First, RSV disease is increasingly recognized as a significant contributor to adult respiratory illnesses worldwide.^5^ Although pregnant women may receive an RSV vaccine to protect her child, other more direct benefits to the mother may be realized. Pregnancy is considered an immunologically attenuated state, and RSV infection during pregnancy is associated with more severe disease and adverse outcomes (e.g., fever, respiratory distress, preterm labor, hospitalization).^6^ As a result, an RSV vaccine could improve maternal as well as infant outcomes and should be considered as part of cost/benefit analyses when planning vaccine introduction. Second, family members, including the mother, may be the source for neonate exposure to RSV; therefore, disease prevalence and modulation of that prevalence in an adult population is germane to this discussion. Third, recent data suggest the possibility of vertical RSV transmission during the prenatal period. When vertical transmission is induced in animal models, specific long-lasting alterations in immunologic and pulmonary functions have been demonstrated in the offspring of mothers acquiring the infection prenatally. Last, the degree of disease burden in adults may influence the timing of RSV vaccination, in that, if the burden is deemed low, the vaccine may be intentionally delayed to later in the third trimester to optimize antibody transfer and newborn protection.

To better understand and characterize RSV disease burden in adults, women of childbearing age, and pregnant women, we performed a literature search using English-language articles identified in the PubMed and Google Scholar databases, with additional resources identified by extraction from references within those documents. Keywords or phrases searched, used separately or in combination were: respiratory syncytial virus, RSV, epidemiology, adult respiratory disease, and pregnancy.

### RSV Biology

Human RSV is a member of the *Paramyxoviridae* family. It is a single stranded RNA virus that has 10 genes encoding 11 proteins. Two of these proteins, F and G, are particularly important for virus attachment and syncytia formation on target host cells. These 2 surface proteins are also the primary target antigens currently being investigated for both active and passive host immunologic protection. RSV is a virus that causes respiratory tract infections with a wide clinical spectrum. Outbreaks of RSV infections occur between fall and spring in temperate climates and tend to last up to 5 months. RSV isolates can be divided into 2 groups: group A and group B, based on antigenic and genetic characteristics. These 2 groups coexist in the human population, with group A being more prevalent. Most studies have not found significant clinical differences between both subtypes.^7^

## RSV IN THE ADULT POPULATION

Data for RSV in the adult population are limited by the paucity and quality of available studies.^8^^–^^14^ Regardless, certain general observations can be gleaned from the current literature and are summarized below. Data from an adult RSV-season surveillance program from 1975 to 1995 examined routinely obtained viral culture specimens and identified an RSV prevalence rate of 7% in the population.^8^ The majority of subjects sampled were between 18 and 40 years old, and 65% were females. Of interest, 84% of culture-positive individuals were symptomatic, with 74% reporting upper respiratory symptoms, 26% with lower respiratory tract findings, and about half noting subjective fever. Mean duration of viral shedding was 3.9 days, with a wide range (1–17 days). Compared to subjects with upper respiratory tract symptoms but who were RSV culture-negative, those with documented RSV infection had more prolonged symptoms and a greater frequency of concomitant lower respiratory findings. The investigators also noted that RSV infection was more commonly associated with acute exacerbations of chronic cardiopulmonary conditions, pulmonary function abnormalities, and increased mortality in immunocompromised hosts, although specific data were not presented. They also remarked on the propensity for RSV to spread among family members and those living in close quarters, although, again, data were not offered.

Data for RSV in the adult population are limited by the paucity and quality of available studies.

Internationally derived data show similar RSV infection rates in adults with respiratory symptoms. O’Shea et al. demonstrated an 11% prevalence of RSV in otherwise healthy male U.S. military recruits presenting with acute respiratory symptoms.^9^ Using several isolation methodologies with different degrees of sensitivity for pathogen identification, these investigators also showed significant differences in detection rates, helping clarify the wide ranges reported in related studies. For example, Zambon et al. studied adults with upper respiratory tract infections in the United Kingdom and demonstrated a 20% RSV positivity rate using a sensitive polymerase chain reaction (PCR) technology.^10^ In contrast, in a study in Kenya of patients with influenza-like illness or other acute respiratory symptoms, Bigogo et al. only detected a 4.2%–6.5% PCR-positive RSV occurrence.^11^ However, this latter study was significantly compromised by an incomplete data collection process. These same investigators noted that adults with HIV infection were twice as likely as non-HIV patients to have RSV identified as a causative agent when presenting with acute respiratory illness.

One study attempted to diagnose RSV infection irrespective of symptoms. Munywoki et al. serially sampled 47 households (n=493) in rural Kenya throughout a complete RSV season regardless of symptomatology and found that 37% of the subjects had at least 1 RSV episode, with 58% symptomatic when RSV-positive by PCR.^12^ Factors associated with symptomatic disease included younger age groups, higher viral loads, and extended shedding times. For individuals aged 15–40 years, 26% were identified to have at least 1 RSV episode, with only 24% being symptomatic. Fever was an uncommon symptom (14%), few sought medical care (16%), and none were hospitalized in the entire study cohort.

### Complications of RSV Infection in Adults

The majority of RSV cases in the adult population are presumed to represent reinfection with this pathogen because primary acquisition in childhood is so common. Although most cases manifest clinically as mild to moderate upper respiratory disease, more severe complications have been recognized to occur. A prospective study in the United States of hospitalized adults with acute respiratory infections during the influenza season showed an RSV PCR prevalence rate of 8.7% in adults 50–64 years old.^13^ Most of these individuals had at least 2 preexisting chronic comorbidities (e.g., congestive heart failure, chronic obstructive pulmonary disease, obesity) identified. In a multi-institutional study in France that examined adults hospitalized with influenza-like illness, 4% were RSV-positive by multiplex reverse transcription-PCR.^14^ Although the majority of these subjects were elderly, making it difficult to compare to a reproductive-age population, there was a 57% complication rate.^14^ Pneumonia was identified in 44%, ICU admission was required for 15%, and the in-hospital case fatality rate was 8%. Luchsinger et al. studied 356 Chilean adults with community-acquired pneumonia and found an RSV prevalence of 13.4% by multiple detection methods including serology.^15^ In 2 reviews of RSV disease and its complications in adults, experts noted that RSV accounts for between 2%–5% of adult pneumonia cases annually with an increase to 5%–15% of cases during the RSV season.^6^^,^^16^ In the United States, it is estimated that between 11,000–17,000 adults die of RSV-related infection annually with 10-fold more admitted to the hospital with respiratory compromise. Han et al. reported the annual economic burden in the United States of adult inpatient care related to RSV approximated $150–680 million, with actual health care cost being much higher if non-pneumonia and outpatient cases were included in this calculation.^17^ There are no similar cost estimates for other developed or low- to middle-income countries, but this will be an important consideration when planning for future interventions such as the introduction of RSV vaccines.

### RSV in Reproductive-Aged Women and Pregnancy

Even fewer data on RSV are available in women of childbearing age or related to pregnancy.^18^^–^^24^ In 2 different phase-2 clinical trials in healthy women 18–35 years old that looked at the potential for RSV vaccine use in reproductive-aged women, investigators reported a 10%–15% Western blot RSV positivity rate at baseline, likely representing previous or recent exposures.^18^^,^^19^ Interestingly, in both of these vaccine trials, which spanned 2 RSV transmission seasons, there was Western blot evidence of new RSV infection in 21% and 22%, respectively, in the placebo-arm study participants, unrelated to clinical symptomatology. In a study focused on influenza in rural Nepal, Chu et al. found a 2% RSV PCR positivity rate in pregnant or recently postpartum women with fever and acute respiratory symptoms.^20^ Similarly, Chaw et al., in another prospective observational study of symptomatic pregnant women in Mongolia during the influenza season, noted a 2.4% RSV positive rate using a point-of-care immunoassay test.^21^ Both of these latter 2 studies were limited by a primary focus on influenza-related symptoms, missed testing times, and other study design issues. In conjunction with a prospective maternal influenza vaccine trial, Madhi et al. retrospectively studied the incidence of RSV positivity by PCRin HIV-negative and HIV-positive South African gravidas and postpartum women.^22^ Women presenting with respiratory illness from mid-pregnancy until 24 weeks postpartum underwent oral/nasal/pharyngeal swab sampling. The incidence of RSV associated illness (cases per 1000 person-months) during pregnancy in 2011 was 1.7 and 6.6 for HIV-negative and HIV-positive women, respectively. The incidence postpartum was identical for these 2 study populations at 2.3. The authors made additional observations, including the fact that RSV infection during pregnancy did not appear to be associated with any adverse pregnancy outcomes compared to the non-RSV infected groups. They expressed that their study likely underestimated the overall prevalence of RSV due to the focus on influenza-related disease, the variable incidence over RSV seasons (they noted the incidence in pregnancy in 2012 was 5.3 for HIV-negative pregnant women), and the fact that most RSV cases were identified during unsolicited medically attended visits which likely missed cases of milder disease. Hause et al. performed an outpatient, cross-sectional, surveillance study in pregnant women in their second and third trimesters in Houston, Texas. Of 81 subjects who presented with acute respiratory illness symptoms during the RSV season, 10% were identified to have RSV using tandem competitive-PCR.^23^

Even fewer data are available on RSV in women of childbearing age or related to pregnancy.

Regarding pregnancy complications associated with RSV infection, and despite the observations mentioned by Madhi et al.^22^ in the study by Chu et al.,^20^ of 7 patients with acute respiratory symptoms, fever, and RSV positivity, 2 (29%) experienced preterm birth. Given the very small numbers, statistical analysis comparing these subjects to the non-RSV infected study population (preterm birth rate 13%) was not possible. In a recent U.S. tertiary care center case report series, Wheeler et al. reported on 3 cases of symptomatic RSV infection during the antepartum period, with 2 of these patients requiring hospitalization and intubation for respiratory failure.^6^ Both of these patients had other comorbidities including smoking and preexisting asthma.

In an international study examining RSV infection in pregnant women from high-income countries who were hospitalized, 48% were severe enough to require prolonged hospitalization and 38% were subsequently diagnosed to have pneumonia. The majority of these RSV cases were detected in the third trimester, and one-third had a preexisting health condition, most commonly asthma. Among women who did not deliver during that admission, there was a 29% preterm birth rate.^24^ Limitations to this study, as is the case with most other studies on this topic, were the small number of patients sampled for RSV, a restriction to hospitalized patients only, and a study design that focused on influenza in pregnancy.

Although tangential in nature, data on influenza in pregnancy, another common viral pathogen, show that pregnant women are 7 times more likely to be hospitalized and 2 times more likely to die compared to non-pregnant women of reproductive age.^25^ Lastly, and also tangential, pregnant women with pneumonia due to various pathogens, have an increased risk for several adverse pregnancy outcomes, including low birth weight, preterm birth and cesarean delivery.^26^ As RSV continues to be investigated in this patient population, these types of adverse events need to be carefully monitored.

A novel concern is the concept of vertical transmission of RSV. Using a rodent model, investigators have recently reported evidence of RSV vertical transmission from mother to fetus, with persistence of infection in the offspring after birth.^27^^,^^28^ These exposed and infected newborn pups showed evidence of prolonged altered viral immunity, dysregulation of neurotrophic pathways, and, most importantly, airway hyper-reactivity with RSV reinfection. Using human specimens, Fonceca and colleagues recently reported rescue of infectious RSV from cord blood mononuclear cells in 26 of 45 (57.7%) samples, with increases in recovery during winter months.^29^ These findings await confirmation, but suggest another avenue of RSV pathogenesis that might be interrupted by preventing maternal disease via vaccination.

## RELEVANCE TO POLICY MAKERS AND PROGRAM MANAGERS IN LOW- AND MIDDLE-INCOME COUNTRIES

Concomitant with ongoing basic and clinical science research exploring maternal RSV vaccination and passive newborn protection, policy makers and program managers need to begin examining the potential impact of these advances on their specific populations and prioritize the use of available resources to accomplish these goals. This effort is particularly germane in low- and middle- income communities where it is anticipated that disease burden is likely underestimated and the lack of existing infrastructures have compromised the collection of this critically needed information. Characterization of temporal and geographic patterns of RSV circulation by country and region will be needed, because this will directly impact the accuracy of disease burden estimates and influence planned interventional strategies. Using prospective active pathogen surveillance with sensitive diagnostic tools across a wide demographic range would be ideal, but these types of platforms will require partnerships with regional health authorities, international agencies, established research units, and global funders. The majority of RSV-related data pertaining to disease severity thus far has been collected in developed countries and in hospitalized or medically attended cases. These same data need to be extended to a broader patient base if accurate morbidity and mortality estimates are to be calculated for less developed countries. Additionally, more refined age group strata, with particular emphasis on extremes in age, will be needed to address the impact of vaccination in specific populations, such as pregnant women, very young children, and the elderly. Adequate data are lacking regarding the impact of RSV disease with specific comorbidities such as HIV disease. The role of environmental factors like smoke and indoor air pollution in RSV pathophysiology also need to be explored. More information on long-term disease outcomes, such as RSV reinfection and chronic wheezing, will help better define cost-effective and quality of life analyses that are critical components when considering the introduction of new therapeutic interventions. Last, if injectable vaccination is the preferred route of administration, a detailed analysis of population health care environments, drug delivery systems and pathways, and available personnel will need to be examined particularly in low-resource settings. A detailed description of this topic, including future research agenda items for consideration, can be found in a recently published document by PATH entitled “Advancing RSV Maternal Immunization: A Gap Analysis Report.”^4^

Policy makers and program managers should examine the potential impact of research advances in maternal RSV vaccination on their specific populations.

## CONCLUSIONS

In summary, this review highlights the limited and poor quality of the currently available surveillance and natural history RSV data in the adult population, particularly in low- to middle-income countries where the disease burden is likely to be significantly greater.^30^ These data, specific to country or region, are critically needed to better understand the full extent of this pathogen’s disease burden and to optimize future vaccine-related interventions. To this latter point, both the World Health Organization and the Bill & Melinda Gates Foundation have spearheaded research efforts to address these deficiencies. Despite significant methodologic concerns with the current data (such as varying approaches to pathogen identification, retrospective use of samples collected for other studies, etc.), it would be reasonable to speculate that the overall prevalence of symptomatic RSV infection in reproductive-aged or pregnant women is low, and severe morbidities are uncommon. Although isolated cases of severe lower respiratory disease in pregnancy have been reported, these instances may have been precipitated by other underlying comorbidities such as chronic lung disease and HIV infection. These potential relationships require further study. There is likely a much larger cadre of mildly symptomatic or asymptomatic pregnant women that remain of interest because of the risk for disease propagation and the potential for vertical transmission with long-term consequences in the progeny. As interventional investigations progress, the pregnant host should not be seen as simply a vehicle for passive antibody delivery to the fetus, but also as a potential beneficiary of active RSV vaccination.^31^

## Notes

**Peer Reviewed**

**First Published Online:** November 27, 2019

**Competing Interests:** Data for this work was in part generated as a result of collaborative efforts with PATH, a global nonprofit health organization based in Seattle, Washington, for the development of a gap analysis for the Advancing Maternal Immunization project. Dr. Gonik is a site principal investigator for an ongoing Phase 3 randomized, placebo-controlled study to determine immunogenicity and safety of an RSV F nanoparticle vaccine in pregnant hosts by Novavax, Inc. (ClinicalTrials.gov identifier: NCT02624947).
